# A study on fast calling variants from next-generation sequencing data using decision tree

**DOI:** 10.1186/s12859-018-2147-9

**Published:** 2018-04-19

**Authors:** Zhentang Li, Yi Wang, Fei Wang

**Affiliations:** 1 Shanghai Key Lab of Intelligent Information Processing, Shanghai, China; 2 School of Computer Science and Technology, Fudan University, Shanghai, China; 3 MOE Key Laboratory of Contemporary Anthropology and State Key Laboratory of Genetic Engineering, Collaborative Innovation Center of Genetics and Developmental Biology and School of Life Sciences, Fudan University, Shanghai, 200438 China

**Keywords:** Next-generation sequencing, Variant calling, Decision tree

## Abstract

**Background:**

The rapid development of next-generation sequencing (NGS) technology has continuously been refreshing the throughput of sequencing data. However, due to the lack of a smart tool that is both fast and accurate, the analysis task for NGS data, especially those with low-coverage, remains challenging.

**Results:**

We proposed a decision-tree based variant calling algorithm. Experiments on a set of real data indicate that our algorithm achieves high accuracy and sensitivity for SNVs and indels and shows good adaptability on low-coverage data. In particular, our algorithm is obviously faster than 3 widely used tools in our experiments.

**Conclusions:**

We implemented our algorithm in a software named Fuwa and applied it together with 4 well-known variant callers, i.e., Platypus, GATK-UnifiedGenotyper, GATK-HaplotypeCaller and SAMtools, to three sequencing data sets of a well-studied sample NA12878, which were produced by whole-genome, whole-exome and low-coverage whole-genome sequencing technology respectively. We also conducted additional experiments on the WGS data of 4 newly released samples that have not been used to populate dbSNP.

## Background

Next-generation DNA sequencing (NGS) technologies have made great progress in both improving throughput and lowering cost in recent years. Today, NGS technology can finish a whole-genome sequencing task in a single day for merely one thousand dollars [1]. The massive data sets generated by NGS in research projects such as 1000 Genomes are counted in terabases [2], and it is predicted that in the next decade, approximately one hundred million to two billion human genomes will be sequenced [1]. Facing challenges from the explosive growth of sequencing data, faster and more efficient data analysis tools are required.

Variant calling is a key link in the NGS data analysis workflow. The quality of call sets directly affects downstream analysis such as disease-causing gene detection. To call variants from sequencing data, an aligner such as BWA should be used to map and align short reads generated by NGS platforms to the reference genome first; then, a variant caller is applied to the aligned results to produce high-quality variant calls as well as genotyping. Early on, tools such as MAQ [3] handled both steps. Since the SAM/BAM format [4] was developed in 2009, researchers were able to concentrate on developing better algorithms for variant calling, leaving out the mapping step. So far, many excellent variant callers have been springing up, including SAMtools [4], Genome Analysis Toolkit (GATK) [2] and Platypus [5].

Variant calling algorithms aim to address technical difficulties such as homopolymer errors, random mutations, insertions and deletions (indels), mis-alignments, and PCR bias. Generally, there are two paradigms [6]. The first paradigm is the Bayesian approach. This paradigm generates candidate variants directly from the results of independently mapping each read to the reference sequence, succeeded by using Bayesian methods to model sequencing errors and identify variants. This paradigm is very powerful for detecting SNVs but may get confused when aligning reads to the region beside candidate indels. The second paradigm is an assembly-based approach. This paradigm first performs de novo assembly of short reads within a fixed-length window to construct candidate haplotypes and then calculates their likelihoods comparing to the reference sequence. The candidate haplotype with the highest likelihood is regarded as the true sequence within that window, and variants contained by that haplotype will be called. This paradigm can address incorrect alignments surrounding indels as well as identify large indels, improving accuracy and recall compared to the first paradigm. However, because of the extremely high computational complexity and huge number of candidate haplotypes, this paradigm requires much a longer runtime. Among the most popular callers, SAMtools and GATK-UnifiedGenotyper [7] follow the first paradigm, while GATK-HaplotypeCaller follows the second paradigm. There is another method that combines the two paradigms, which can also be considered a Bayesian haplotype method, including FreeBayes, PyroHMMvar and Platypus.

However, there are two main shortcomings of the paradigms mentioned above: first, they are not fast enough (as will be shown in our experiments); second, they cannot easily adapt variations in input data type, such as low-pass sequencing data, because they have many default parameters that are difficult to adjust for non-experts. To find another way, some researchers have set their sights on machine learning, such as SNooPer [8], which is a random-forest-based somatic variant caller. SNooPer’s variant detection procedure involves two phases: in the training phase, it trains a random forest model from an orthogonally validated dataset; and in the calling phase, it generates candidate variants and calculates related features from inputted mpileup files and then applies the trained model to classification. As is known, the prediction ability of machine learning algorithms heavily depends on the size and representativeness of the training set. To ensure that machine learning algorithms work well, the training set must be carefully selected. The largest and most authoritative dataset of SNVs and indels is the single nucleotide polymorphism database (dbSNP) [9]. It is reported that over 90% of human genome SNVs and indels have been catalogued in dbSNP [7], so we have confidence in hypothesizing that an unreported variant should be somehow similar to those in dbSNP if it is a true positive and distinct if it is a false positive. Based on this hypothesis, we propose a new method that trains a decision tree from dbSNP and candidate variant set, merging the training and calling phases into one step so that the time cost can be significantly reduced, while other key indicators such as accuracy and recall also have satisfactory results in our experiments.

We have implemented our algorithm in a programme named “Fuwa”. Comparison with 4 currently popular variant callers indicates that when processing whole-genome sequencing data, Fuwa is obviously faster than its competitors, while other key performance indicators also improve or stay comparable, even for variants not in dbSNP. For processing exome-capture and low-pass sequencing data, Fuwa also shows its outstanding capability and flexibility for data type diversity.

## Methods

### Overview of Fuwa

**Fig. 1:**
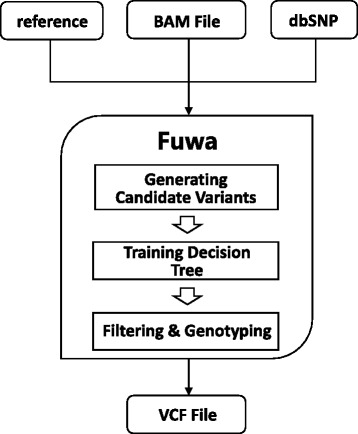
Workflow of Fuwa. Fuwa is designed to translate single BAM file into high quality variants calling output in VCF format. At first, aligner such as BWA maps reads to reference genome and provides BAM file to Fuwa. Then, at each locus of genome, candidate variants are generated from the CIGAR field of piled up reads covering that locus. Each candidate variant is assigned a 0/1 value named dbSNP quality (qual), according to whether it is included in dbSNP. Next, the candidate set is used to build a decision tree. After the tree is build, qual values of variants in the same leaf will be replaced with the average qual value of that leaf. Finally, Candidate variants with low qual (default threshold 0.8) are filtered out, while the rest are called and genotyped. Final call set is output in VCF format

Fuwa accepts single sample alignment data in Binary Sequence Alignment/Mapping (BAM) format and outputs calls for SNVs and short indels in Variant Call Format (VCF) [10]. As shown in Fig. 1, the workflow of Fuwa can be divided into three phases: candidate variants generating, decision-tree building, and variant calling. First, the programme generates candidate variant set by pile-up at each candidate variant locus marked by the CIGAR field. Each candidate variant is marked with a quality metric “qual” valuing 1 or 0 according to whether the candidate variant is in dbSNP. Then, a decision-tree model is trained using the feature vectors of candidate variants as the training set. After the model is trained, candidate variants with similar feature values are grouped into a same leaf node and are treated as a unit. For all the candidates in a leaf, if their average qual is higher than the threshold, they are called out; otherwise, they are identified as false positives. Finally, a simple and effective genotyper is applied.

### Generating and labelling candidate variants

Fuwa walks through the whole-genome sequence, generating candidate variants at each locus. Designed for high sensitivity, Fuwa considers all 6 possible candidate variants (i.e., A, T, G, C, insertion, deletion), and only those with too low a proportion of read depth at their loci are excluded. Feature values of these candidates are also calculated. At the same time, the programme searches dbSNP and labels each candidate with dbSNP quality, or “qual” in short. Qual is set to 1 if the candidate exists in dbSNP and 0 if not. To improve search speed, Fuwa preloads dbSNP into RAM and transforms it into a hash table so that any searching can be finished in a constant time. After this step, all candidate variants are obtained and labelled.

To date, most common human variants have already been catalogued in dbSNP. The high coverage rate of SNVs and short indels qualifies dbSNP as a powerful benchmark in alignment result recalibration [7] and final call set quality assessment [5, 7, 11] as well as in training machine learning models.

### Decision tree and feature selection

Classification and regression tree (CART) [12] is a widely used training algorithm of decision tree that can be applied to either classification or regression problems. It assumes the decision tree to be binary, and each non-leaf node is measured by a Boolean expression so that the input samples could be transferred into two branches: the left branch if the Boolean expression is “true” or the right branch otherwise. We chose CART because it is simple and fast, and the decision procedure can be easily understood.

Twelve features were selected to train the CART model, which were divided into four categories, shown as follows.

#### Category I. Read depth

Features under this category measure the absolute depth and depth ratio of reads that are “effective” to be a specific candidate variant. “Effective” means that the read shares the same base as the candidate variant at the candidate’s locus.

##### Feature 1: effective base depth

Effective Base Depth (EBD) is the sum of the depths of effective reads. For indel reads, the EBD equals the mapping quality, while for SNV reads, the EBD is the value of the mapping quality multiplied by the base quality.

##### Feature 2: effective base depth ratio

The EBD ratio, i.e., the EBD of one candidate variant divided by the sum of the EBDs of all candidate variants at that locus. If this indicator is very low, the related candidate variant tends to be a random error.

##### Feature 3: DeltaL

DeltaL is a statistic describing the difference between optimal and suboptimal genotypes. Fuwa first hypothesizes that the variant is true, so the reads covering this locus obey an almost ideal variant model: 0/1 or 1/1. The logarithms of likelihood under these two ideal models are calculated separately, and the bigger one is selected as *L*_1_. Then, Fuwa calculates the second likelihood logarithm, *L*_2_, under another hypothesis that the variant is false and that reads covering this locus follow the binomial distribution model. Thus, *L*_1_*-L*_2_, or DeltaL, is the logarithm of the ratio of the first and second likelihoods. If DeltaL is close to 0, which means the likelihoods of the ideal model and the binomial model are nearly equal, we empirically judged the variant to be false positive; otherwise, the variant tends to be true.

#### Category II. Base quality

This category focuses on the accuracy of a base sequenced by the sequencing machine, which has considerable impact on variant calling.

##### Feature 4: Sum of Base Quality (SumBQ)

This feature is the sum of the base quality of effective reads for one candidate variant. For indel reads, this value is set to 30 empirically.

##### Feature 5: Average Mapping Quality (AveBQ)

By dividing SumBQ by the number of effective reads, we obtain the average mapping quality.

##### Feature 6: Variance of Position (VarPos)

Here, “position” means the offset of the pile-up site from the 3′ end of a read. We use this statistic considering that, generally, sequencing quality declines towards the end of a read; thus, candidate variants that are close to the 3′ end are more likely to be sequencing errors.

#### Category III. Mapping/alignment quality

This category considers how well a read is mapped and aligned to its current locus. Mismatches lead to a higher possibility of false positives.

##### Feature 7: Average Mapping Quality (AveMQ)

The average of the mapping quality of effective reads at the candidate variant’s locus.

##### Feature 8: Worst Mapping Quality (WorMQ)

The worst mapping quality of all reads at the candidate variant’s locus.

##### Feature 9: Poor Mapping Quality Ratio (PoorMQR)

The ratio of reads with mapping quality lower than 15 at the candidate variant’s locus.

##### Feature 10: Average Alignment Score (AveAS)

The alignment score is a different metric than mapping quality, and its computing methods vary from aligner to aligner. Briefly speaking, the alignment score measures the similarity between a read and the reference genome, while mapping quality reflects the specificity that a read tends to be mapped to its current locus instead of other loci. AveAS is the average of the alignment scores of all reads at the candidate variant’s locus.

#### Category IV. Strand Bias

This category assumes that effective reads of true positives from positive and negative strands of DNA should be approximately equal.

##### Feature 11: Variance of Strands (VarStr)

Assuming that the numbers of effective reads from positive/negative strands obey the binomial distribution, the variance can be calculated through the formula *D*(*n*) *= np*(1*-p*). If VarStr is small, it means that reads of the candidate variant cluster in one direction, suggesting a sequencing error or other false positive situations.

##### Feature 12: Bias of Strands (BiasStr)

BiasStr is a *χ* ^2^ value measuring the significance of correlation between “whether a read is effective” and the direction of strand that the read comes from. It is calculated by using a 2 × 2 contingency table (see Table 1):

$$ {x}^2=\frac{n{\left( ad- bc\right)}^2}{\left(a+b\right)\left(c+d\right)\left(a+c\right)\left(b+d\right)} $$

where *n = a* + *b* + *c* + *d*.

**Table 1:** Contingency table for calculating BiasStr

Strand Direction Effective	Positive	Negative
Yes	a	b
No	c	d

If BiasStr is too high, which means the effective reads of the candidate variant cluster in one strand, the candidate tends to be caused by sequencing error.

### Modelling, calling and genotyping

When the training set is ready, Fuwa trains a decision tree using CART training algorithm. Once the tree is built, all candidate variants in each leaf node are assigned a new qual value, which is the mean qual of all candidate variants in that leaf node. Candidates with a qual higher than the threshold are reported as true variants in the final call set. The default threshold is set to 0.8 for SNPs and 0.6 for indels empirically.

Fuwa adopts a simple but effective genotyping strategy: if the effective depth of alternative reads is more than ten times the effective depth of reference reads, the genotype is considered homozygosity; otherwise, it is considered heterozygosity. This strategy is sufficient for most demands, and more precise (also slower) genotyping methods such as population-based genotyping can be applied if needed.

## Results

### Application 1: calling variants from whole-genome, exome-capture and low-coverage whole-genome sequencing data of NA12878

A well-studied sample, NA12878 (CEU cohort from Utah of northern and western European ancestry) from the 1000 Genomes Project [13], was analysed to evaluate the performance of Fuwa. We started from HiSeq WGS (75~ 86× 101-bp paired-end) data, exome-capture (average 210× 100-bp paired-end) data and low-coverage (~ 4×) whole-genome sequencing data, conducted read alignment with BWA (version 0.7.12), and applied preprocessing steps including duplicate removal, local realignment and base quality recalibration before the calling step. After the call sets were generated, we used the Axiom chip, high-quality haploid fosmid data and the NIST Genome in a Bottle integrated calls v0.2 (GIAB) [14] as benchmarks to evaluate these call sets. We compared Fuwa to 4 well-known DNA variant callers: SAMtools, GTAK-UnifiedGenotyper, GATK-HaplotypeCaller and Platypus, using all their latest version (SAMtools 1.3.1, GATK 3.7, and Platypus 0.8.1), default settings and applying their official “best practices”. We noticed that GATK 4 just released a beta version. In GATK 4, UnifiedGenotyper has been removed, while HaplotypeCaller for germline variants is directly inherited from GATK 3.7, and the experimental results of HaplotypeCaller from GATK 3.7 and GATK 4 are very close.

### Calling variants from HiSeq whole-genome data

The experimental result indicates that Fuwa achieves fast speed and high precision in calling both SNVs and indels, with no obvious shortcomings (Table 2). The transition /transversion ratio of 2.03 is close to that in a previous study [15], which suggests good specificity for SNVs. Axiom SNP chip data offered strong support: Fuwa achieved the highest genotype concordance (99.32%) and lowest mono rate (0.04%). Although Fuwa called 3,820,377 SNVs, which was not as many as GATK-UnifiedGenotyper (4441130), GATK-HaplotypeCaller (4034309) or SAMtools (3959135), its recall against Axiom data (96.81%) and fosmid data (93.5%) is close to the three callers mentioned above.

**Table 2:** Comparison of four variant callers on whole-genome sequencing data

		Whole genome				
		Fuwa	Platypus	GATK-UG	GATK-HC	SAMtools
SNPs		3,820,377	3,271,282	4,441,130	4,034,309	3,959,135
Ti/tv		2.03	2.13	1.84	1.94	2.01
Axiom	GT concordance (%)	99.32	98.29	97.3	98.52	99
	Sensitivity (%)	96.81	94.34	97.41	97.16	96.88
	Mono rate (%)	0.04	0.13	0.22	0.11	0.07
Fosmid	Recall (%)	93.5	90.7	95.03	94.56	93.79
GIAB	Recall (%)	98.41	89.34	98.65	98.44	97.89
	Precision (%)	99.26	99.69	97.72	98.79	99.47
Indels		649,387	575,350	711,045	884,204	765,800
In-frame fraction		0.47	0.47	0.46	0.51	0.45
Fosmid	Recall (%)	68.04	75.69	64.31	72.25	60.59
GIAB	Recall (%)	87.48	69.49	89.74	94.7	80.98
	Precision (%)	95.93	78.49	95.59	94.08	92.32
Runtime (real time, min)		127	233	1058	2545	1546

Ti/tv, transition/transversion rate; GT concordance, concordance of genotypes at Axiom-called loci; Sensitivity, ratio of non-reference calls at Axiom-called loci; Mono rate, fraction of monomorphic Axiom sites that are called as variants; In-frame fraction, fraction of indels (limited to coding regions) whose length are integer multiples of 3; Runtime, CPU minutes needed to process the input bam file; Recall = TP/(TP + FN); Precision = TP/(TP + FP); *TP* true positive, *FN* false negative, *FP* false positive

Using orthogonal technology such as Axiom and fosmid to estimate quality metrics has many limitations because microarray sites are not randomly distributed among the whole genome, as they only have genotype content with known common SNVs in regions that can be accessed by the technology. To overcome these limitations, we introduced the integrated call set of NA12878 from the Genome in a Bottle Consortium as benchmark, which combines 14 data sets from 5 sequencing technologies, 7 read mappers, and 3 variant callers: GATK-UnifiedGenotyper, GATK-HaplotypeCaller and Cortex. The source of the GIAB data suggests this benchmark in favour of GATK and may not be friendly to new callers. However, Fuwa still performs well: both recall and precision of GIAB are only slightly lower than the best values of corresponding metrics, further providing powerful evidence of Fuwa’s high sensitivity and accuracy on SNV calling in genome-wide data.

Indel calling is a more challenging task than SNV calling, but Fuwa can also perform well at this task. Frameshift indels in coding regions of DNA nearly always lead to the loss of function of proteins, so the frameshift fraction of indels is considered to be lower in coding regions than in non-coding regions. A previous study showed that approximately 50% of coding indels cause frameshift [16]. In the results of NA12878 whole-genome data calling, Fuwa called 649,387 indels with an in-frame fraction (fraction of indels that do not lead to frameshift) of 0.47, indicating high quality of the call set. Fuwa achieves the highest precision on GIAB (95.93%), while its recalls against fosmid data (68.4%, average 68.18%) and GIAB (87.48%, average 84.48%) are acceptable; from these data, we can estimate a low false-positive rate. Platypus achieved the highest fosmid recall (75.69%) with the smallest call set size (575350), which made it appear to have the highest precision, but indicators from GIAB showed the opposite result. We infer that this situation occurred because the fosmid chip only covers a small number of sites (1057) and the algorithm of Platypus may be more specific for these sites than other callers.

To evaluate Fuwa’s ability to call variants not in dbSNP, we excluded variants that are in dbSNP from Fuwa, Axiom, Fosmid, and the 1000 Genomes call sets, and then we recalculated the same metrics. The results are shown in Table 3. Specifically, Axiom called 299 non-reference sites, and Fuwa rediscovered 289 of them; Fosmid called 495 variants, and Fuwa rediscovered 315 of them; the 1000 Genomes confident call set contains 285,095 variants not in dbSNP, and Fuwa called 251,095 of them. We observed that Fuwa can still predict most variants, indicating that Fuwa has gained power to infer new variants through the model training process. Thus our basic assumption that, real variants not in dbSNP and variants in dbSNP should have similar characteristics for the 12 features, is supported.

**Table 3:** Comparison of Fuwa’s callsets on NA12878 WGS data before and after variants in dbSNP are removed

		All	non-dbSNPs
Axiom	GT concordance(%)	99.32	100.00
	Sensitivity(%)	96.81	96.66
	Mono Rate(%)	0.04	0.00
Fosmid	Recall(%)	88.11	63.63
1KG confident call set	Recall(%)	95.31	88.07

Since calling rare variants is the challenging but yet important component, we specifically evaluated Fuwa’s ability to call rare variants. According to Table 4, we estimated that Fuwa’s sensitivities for variants with an allele frequency lower than 5% (73.21%), 1% (62.87%), 0.5% (60.26%) and 0.1% (63.08%) are very similar to those of Platypus, GATK and SAMtools (average 73.19%, 62.77%, 60.12% and 62.87%). Further study showed a high coincidence of the rare variants (AF ≤ 5%) callsets of the 4 callers, specifically over 99% rare variants called by Fuwa are also called by GATK, suggesting good specificity of Fuwa for calling rare variants.

**Table 4:** Comparison of four variant callers for calling rare variants

AF		benchmark (high-conf)	Fuwa	Platypus	GATK-UG	GATK-HC	SAMtools
≤5%	count	282,869	207,098	201,577	210,190	209,119	207,187
	ratio (%)	–	73.21	71.26	74.31	73.93	73.24
≤1%	count	128,661	80,895	78,908	81,758	81,417	80,956
	ratio (%)	–	62.87	61.33	63.55	63.28	62.92
≤0.5%	count	92,309	55,630	54,280	56,137	55,928	55,646
	ratio (%)	–	60.26	58.80	60.81	60.59	60.28
≤0.1%	count	37,563	23,695	23,112	23,863	23,795	23,688
	ratio (%)	–	63.08	61.53	63.53	63.35	63.06

*AF* allele frequency

As for run time, Fuwa only spends approximately 2 h (127 min) on the calling process and reduces the CPU time cost by an order of magnitude when compared with GATK (UnifiedGenotyper 1058 min, HaplotypeCaller 2545 min) or SAMtools (1546 min) and by nearly half when compared with Platypus (233 min). The ultra-fast calling speed allows Fuwa to achieve high throughput.

### Calling variants from exome-capture data

Exome-capture sequencing is more efficient and cost-effective than whole-genome sequencing because the time and monetary costs of exome-capture sequencing are much lower than those of whole genome sequencing, and most clinically explicable variants occur in coding regions. We called exome-capture data of NA12878, and then used SNP chips and GIAB integrated calling set to evaluate the sensitivity and accuracy of callers. The analysis results are shown in Table 5. Note that the computation of all the metrics in this table was limited in the coding regions.

**Table 5:** Comparison of four variant callers on whole-exome sequencing data

		Whole exome				
		Fuwa	Platypus	GATK-UG	GATK-HC	SAMtools
SNPs		22,119	21,260	24,777	21,774	20,938
Ti/tv		2.65	2.97	2.36	2.59	2.7
Axiom	GT concordance (%)	96.82	92.83	90.83	95.65	97.15
	Recall (%)	91.09	86.55	92.37	90.34	89.8
	Mono rate (%)	0.1	0.28	0.37	0.16	0.08
Fosmid	Recall (%)	NA	NA	NA	NA	NA
GIAB	Recall (%)	87.59	77.39	87.02	86.37	86.78
	Precision (%)	98.44	99.88	93.06	96.28	97.61
Indels		478	773	405	440	680
In-frame fraction		0.39	0.28	0.35	0.44	0.35
Fosmid	Recall (%)	NA	NA	NA	NA	NA
GIAB	Recall (%)	64.41	51.42	55.35	64.47	52.37
	Precision (%)	92.79	68.87	91.71	96.4	78.24
Runtime (real time, min)		13.5	9.8	93.6	170.5	85.3

*NA* not available. Fosmid call set failed to act as a benchmark on exome data analysis results because it rarely covers sites of exome regions

As shown in Table 5, the overall results are quite similar to those of whole-genome data. Fuwa ranks first in SNV recall against GIAB (87.59%) and second in all other quality metrics, among which most are very close to the best values of the same rows: Axiom genotype concordance (0.33%), Axiom mono rate (0.02%), GIAB SNP precision (0.44%) and GIAB indel recall (0.06%), indicating good specificity for exome sequencing data. Again, Fuwa finished variant calling process at time cost of an order of magnitude less than that of GATK and six-sevenths less than that of SAMtools. Although Platypus ran somewhat (4 min) faster than Fuwa, it produced the worst results for half of the metrics. Overall, Fuwa achieves high speed with a well-balanced performance with regard to accuracy and recall, making it a good choice for exome-capture data analysis.

### Calling variants from low-coverage sequencing data

Low-coverage data pose a great challenge for variant detection because there may not be enough reads at each locus for making the right judgement. To evaluate the 5 calling algorithms’ adaptation for such kind of data, we applied them to NA12878 low-coverage sequencing data (average ~ 4×). The results are shown in Table 6. Consequently, Fuwa’s performance is stable compared to experiments with WGS data and exome-capture sequencing data. Some callers encounter a much sharper reduction in some aspects of performance than others, such as Platypus for SNV recalls (12%~ 17% below average) and GATK-UnifiedGenotyper for indel discovery (4 indel metrics of GATK-UG rank last); these reductions do not occur with Fuwa. In contrast, Fuwa ranks first or second in 7 of 11 comparable items, while the performance on the remaining 4 items is higher or slightly lower than the average level.

**Table 6:** Comparison of four variant callers on low-coverage WGS data

		Low coverage				
		Fuwa	Platypus	GATK-UG	GATK-HC	SAMtools
SNPs		3,023,581	2,233,580	3,121,470	2,494,546	2,846,019
Ti/tv		2.02	2.08	1.95	1.98	1.99
Axiom	GT concordance (%)	92.94	94.25	92.02	93.28	91.29
	Recall (%)	75.88	57.74	77.22	62.89	72.58
	Mono rate (%)	0.03	0.01	0.05	0.02	0.03
Fosmid	Recall (%)	77.68	59.62	78.45	65.59	73.87
GIAB	Recall (%)	79.09	51.32	66.87	66.99	75.78
	Precision (%)	98.94	99.62	99.41	99.48	99.37
Indels		428,290	441,861	108,233	340,404	386,158
In-frame fraction		0.41	0.43	0.37	0.43	0.48
Fosmid	Recall (%)	42.75	42.45	12.75	33.73	35.88
GIAB	Recall (%)	59.33	46.62	16.77	48.5	52.69
	Precision (%)	95.2	79.9	97.79	95.8	94.06
Runtime (real time, min)		37.7	24	138	427.8	312.3

**Fig. 2:**
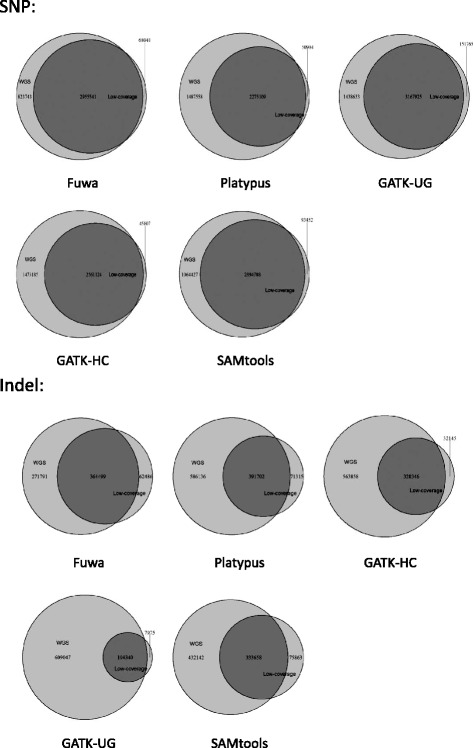
Overlap between WGS high-coverage and low-coverage call sets

To further measure Fuwa’s specificity for low-coverage data, we compared the overlap of call sets of WGS high-coverage and low-coverage data (Fig. 2) for each caller. The Venn diagrams in Fig. 2 indicate that the call sets of Fuwa have a significantly higher overlap ratio against the union set both for SNVs (76.82%) and indels (52.16%) than other callers. The Venn diagram of SAMtools SNV looks similar to that of Fuwa, but its overlap ratio is actually 71.43%, lower than that of Fuwa by 5.39%. For indel, the difference is even more obvious: the second-ranking overlap ratio, which is also from SAMtools, is 39.64%, dropping 12.52% below the value of Fuwa. The result supports that Fuwa has outstanding specificity for low-pass data.

### Application 2: calling variants from data which have not been used to populate dbSNP

Due to the fact that NA12878 has been well studied and almost all of its variants are in dbSNP, we conducted additional experiments on 4 other samples to further evaluate Fuwa’s performance under more general conditions. Three of these samples (NA24149, NA24143, and NA24385) are an Ashkenazim trio and the other one (NA24631) is a Chinese male. These samples are newly released by GIAB and have not been used to populate dbSNP. We used the high-confidence callsets of these samples provided by GIAB as benchmarks for estimating sensitivities of Fuwa and other callers. About 8% variants in these benchmarks are not in dbSNP. The analysis results are shown in Table 7. The results show that Fuwa is a top hunter for SNPs (highest recall 99.91%, highest precision 84.92%), while its ability for calling indels (highest recall 93.52%, highest precision 60.87%) stay comparable to other callers. Although Fuwa is somehow weaker in discovering more indels, its specificity for indel calling is often the highest.

**Table 7:** Comparison of SNP and indel calls on the WGS data of the Ashkenazim Trio and the Chinese sample for the four callers

	benchmark	Fuwa	Platypus	GATK-UG	GATK-HC	SAMtools
a. NA24149						
Total	3,600,577	4,596,629	4,936,516	5,078,361	4,962,252	5,121,162
SNP	3,062,103	3,773,197	3,741,864	4,222,373	4,073,476	4,052,727
Indel	538,474	823,432	1,194,652	855,988	888,776	1,068,435
SNP Recall(%)	–	99.64	94.10	99.88	99.88	99.55
SNP Precision(%)	–	80.86	77.00	72.44	75.08	75.22
Indel Recall(%)	–	91.99	97.26	95.51	96.66	80.30
Indel Precision(%)	–	60.16	43.84	60.08	58.57	40.47
b. NA24143						
Total	3,638,487	4,683,584	5,047,869	5,185,325	5,069,960	5,231,986
SNP	3,089,689	3,848,083	3,818,763	4,304,521	4,153,126	4,127,152
Indel	548,798	835,501	1,229,106	880,804	916,834	1,104,834
SNP Recall(%)	–	99.65	94.12	99.89	99.90	99.55
SNP Precision(%)		80.01	76.15	71.70	74.32	74.53
Indel Recall(%)	–	91.86	97.29	95.73	96.92	80.38
Indel Precision(%)		60.34	43.44	59.64	58.01	39.92
c. NA24385						
Total	3,650,031	4,765,697	4,425,266	4,839,691	4,685,838	5,191,731
SNP	3,101,709	3,942,411	3,452,047	3,987,637	3,803,199	4,123,595
Indel	548,322	823,286	973,219	852,054	882,639	1,068,136
SNP Recall(%)	–	99.91	88.60	99.88	99.92	99.67
SNP Precision(%)		78.60	79.60	77.69	81.49	74.97
Indel Recall(%)	–	91.39	90.14	95.98	98.21	81.96
Indel Precision(%)		60.87	50.79	61.77	61.01	42.07
d. NA24631						
Total	3,655,030	4,599,648	4,935,176	4,987,136	4,871,278	4,667,766
SNP	3,195,050	3,743,038	3,647,691	4,079,102	3,901,018	3,900,109
Indel	459,980	856,610	1,287,485	908,034	970,260	767,657
SNP Recall(%)	–	99.48	93.98	99.93	99.92	99.67
SNP Precision(%)		84.92	82.32	78.27	81.84	81.65
Indel Recall(%)	–	93.52	97.53	97.53	99.54	12.48
Indel Precision(%)		50.22	34.84	49.41	47.19	7.48

We compared the ability of the four callers to call rare and novel variants as is shown in Tables 8 and 9. The results of calling variants from the four samples are all very similar, so for convenience we will take the data of Tables 8a and 9a respectively in the following. We still used high-confidence callsets provided by GIAB as benchmarks, and the values of allele frequencies were obtained from gnomAD. The results in Table 8a show that Fuwa discovered over 98.63% known rare variants of the high-confidence callsets, which is higher than Platypus (95.57%) and is very close to GATK (99.51%). Such results provided more evidence of Fuwa’s specificity for calling rare variants. Meanwhile, we noticed that Fuwa performed weaker than GATK and Platypus in calling variants that are not in gnomAD. Further study showed that Fuwa found about 95.4% non-gnomAD SNPs, which is close to GATK (about 96.2%). But indels are the majority of non-gnomAD variants (average ratio 89.5%) and Fuwa found only 87.8% of them. In Table 9 we compared the performance of the four calling programmes on non-dbSNP variants. The results showed that Fuwa has the highest precisions for both SNPs (78.03%) and indels (31.33%), a very high recall for SNPs (99.26%) and a higher recall for indels (78.23%) than SAMtools. Considering that more sensitive indel calling requires much more complex algorithms and Fuwa achieved such specificities and sensitivities at much higher speed than other callers (see below), we think the weaker performance of Fuwa on discovering novel indels are acceptable.

**Table 8:** Rare and novel variants called by each of the four callers from the WGS data of the Ashkenazim Trio and the Chinese sample

AF		benchmark	Fuwa	Platypus	GATK-UG	GATK-HC	SAMtools
a. NA24149							
≤ 5%	Count	182,482	179,986	174,392	181,248	181,586	179,514
	Recall (%)	–	98.63	95.57	99.32	99.51	98.37
≤ 1%	Count	72,585	71,636	69,736	72,098	72,090	71,434
	Recall (%)	–	98.69	96.07	99.33	99.32	98.41
≤ 0.5%	Count	54,448	53,757	52,379	54,096	54,041	53,603
	Recall (%)	–	98.73	96.20	99.35	99.25	98.45
≤ 0.1%	Count	32,831	32,473	31,607	32,637	32,567	32,402
	Recall (%)	–	98.91	96.27	99.41	99.20	98.69
= 0% (novel)	Count	298,984	265,501	285,754	282,165	282,348	211,935
	Recall(%)	–	88.80	95.58	94.37	94.44	70.89
b. NA24143							
≤ 5%	Count	189,494	187,013	180,969	188,308	188,644	186,607
	Recall (%)	–	98.69	95.50	99.37	99.55	98.48
≤ 1%	Count	74,701	73,773	71,681	74,253	74,215	73,646
	Recall (%)	–	98.76	95.96	99.40	99.35	98.59
≤ 0.5%	Count	55,428	54,744	53,241	55,104	55,023	54,664
	Recall (%)	–	98.77	96.05	99.42	99.27	98.62
≤ 0.1%	Count	33,113	32,711	31,816	32,918	32,831	32,705
	Recall (%)	–	98.79	96.08	99.41	99.15	98.77
= 0% (novel)	Count	304,746	269,764	291,681	288,547	288,978	215,956
	Recall(%)	–	88.52	95.71	94.68	94.83	70.86
c. NA24385							
≤ 5%	Count	187,589	185,929	163,225	186,412	186,909	185,048
	Recall (%)	–	99.12	87.01	99.37	99.64	98.65
≤ 1%	Count	74,132	73,539	64,827	73,693	73,720	73,241
	Recall (%)	–	99.20	87.45	99.41	99.44	98.80
≤ 0.5%	Count	55,153	54,748	48,305	54,851	54,811	54,551
	Recall (%)	–	99.27	87.58	99.45	99.38	98.91
≤ 0.1%	Count	32,722	32,516	28,673	32,547	32,487	32,443
	Recall (%)	–	99.37	87.63	99.47	99.28	99.15
= 0% (novel)	Count	303,456	267,728	259,920	289,030	294,058	222,433
	Recall(%)	–	88.23	85.65	95.25	96.90	73.30
d. NA24631							
≤ 5%	Count	241,718	239,035	230,265	240,706	241,089	222,774
	Recall (%)	–	98.89	95.26	99.58	99.74	92.16
≤ 1%	Count	112,774	111,568	108,087	112,284	112,308	104,437
	Recall (%)	–	98.93	95.84	99.57	99.59	92.61
≤ 0.5%	Count	88,884	87,972	85,370	88,521	88,467	82,493
	Recall (%)	–	98.97	96.05	99.59	99.53	92.81
≤ 0.1%	Count	47,358	46,889	45,557	47,135	47,045	44,078
	Recall (%)	–	99.01	96.20	99.53	99.34	93.07
= 0% (novel)	Count	231,303	208,579	220,259	224,747	227,952	66,392
	Recall(%)	–	90.18	95.23	97.17	98.55	28.70

AF, allele frequency; novel, the variant is not in gnomAD

**Table 9:** Recalls and precisions of the four callers for non-dbSNPs

		benchmark	Fuwa	Platypus	GATK-UG	GATK-HC	SAMtools
a. NA24149							
non-dbSNPs	SNP	235,880	300,076	473,231	644,589	568,941	543,132
	Indel	41,779	104,329	398,110	125,147	158,748	416,411
non-dbSNPs in benchmark	SNP	235,880	234,135	230,167	235,362	235,506	234,330
	Recall (%)	–	99.26	97.58	99.78	99.84	99.34
	Precision(%)	–	78.03	48.64	36.51	41.39	43.14
	Indel	41,779	32,682	37,472	34,926	38,036	29,766
	Recall(%)	–	78.23	89.69	83.60	91.04	71.25
	Precision (%)	–	31.33	9.41	27.91	23.96	7.15
b. NA24149							
non-dbSNPs	SNP	235,889	306,323	486,914	659,433	582,136	551,555
	Indel	42,960	105,352	412,520	131,903	167,074	437,377
non-dbSNPs in benchmark	SNP	235,889	233,881	230,076	235,251	235,477	234,184
	Recall (%)	–	99.15	97.54	99.73	99.83	99.28
	Precision(%)	–	76.35	47.25	35.67	40.45	42.46
	Indel	42,960	33,262	38,642	36,128	39,354	30,455
	Recall(%)	–	77.43	89.95	84.10	91.61	70.89
	Precision (%)	–	31.57	9.37	27.39	23.55	6.96
c. NA24385							
non-dbSNPs	SNP	235,666	337,194	405,643	420,580	335,917	510,292
	Indel	43,316	101,414	287,205	111,591	133,827	393,173
non-dbSNPs in benchmark	SNP	235,666	234,975	216,620	235,010	235,227	234,147
	Recall (%)	–	99.71	91.92	99.72	99.81	99.36
	Precision(%)	–	69.69	53.40	55.88	70.03	45.88
	Indel	43,316	32,730	32,728	36,766	40,921	31,719
	Recall(%)	–	75.56	75.56	84.88	94.47	73.23
	Precision (%)	–	32.27	11.40	32.95	30.58	8.07
d. NA24631							
non-dbSNPs	SNP	238,105	279,070	339,514	426,948	353,943	344,006
	Indel	29,923	108,105	436,409	124,443	160,437	491,021
non-dbSNPs in benchmark	SNP	238,105	236,336	232,361	237,545	237,664	236,804
	Recall (%)	–	99.26	97.59	99.76	99.81	99.45
	Precision(%)	–	84.69	68.44	55.64	67.15	68.84
	Indel	29,923	22,869	26,652	26,007	28,970	6885
	Recall(%)	–	76.43	89.07	86.91	96.82	23.01
	Precision (%)	–	21.15	6.11	20.90	18.06	1.40

Finally, we compared the time, RAM and CPU costs of the four callers when calling NA24149 in Table 10 (the hardware and system environments for experiments are listed in Table 11). Fuwa finished the task in an hour and a half, while Platypus spent half a day, and the slowest caller, GATK-HC, ran two days and a half. Fuwa achieved such a high speed using only one CPU thread and no more than 1.6G RAM, saving much CPU and RAM resources compared to GATK. Moreover, when calling variants from NA24385 (BAM size 284GB after preprocessing), Fuwa finished in 3 h, but GATK predicted itself to run over 8 days, so we had to split the BAM file into 4 ones and ran 4 GATK processes to call them in parallel, each process with 8 threads. Even so GATK still fell behind Fuwa by about 10 h. With the fast increase of the size of single NGS data file, the advantages of Fuwa will be more prominent.

**Table 10:** Runtime comparison of the four calling programs using NA24149 WGS data as input

	Fuwa	Platypus	GATK-UG	GATK-HC	SAMtools
Time(min)	96.97	796	1434.6	3617.4	3025.68
RAM max(M)	1638.4	3174.4	4710.4	6656	284
RAM average(M)	1299.85	1217.72	1092.03	1935.76	192.67
CPU max(%)	100	100	257.2	1336.1	100
CPU average(%)	98.9	98.57	104.23	112.21	98.85

**Table 11:** Hardware and system environments for the experiment

RAM	64GB
CPU	2 physical CPUs, each with 8 cores
CPU model	2.60GHz Intel(R) Xeon(R) CPU E5–2670
Logical Processor	32
OS	Ubuntu14.04.5 LTS x86_64
Java	version 1.8.0_121, Java(TM) SE Runtime Environment

## Discussion

The following command line is a typical invocation of Fuwa:

*fuwa -i sample.bam -d dbsnp141.gz -r reference/ -o sample.fuwa.*

The input file dbsnp141.gz provides variants in dbSNP, and the input directory reference provides all the DNA reference sequences. To cut down the cost of I/O operations and the memory usage, we divided hs37d5.fa into multiple files according to chromosomes, and these files must be put in the same directory, i.e., the reference directory.

In a single run, besides the VCF file Fuwa also outputs a “tree” file that records all the nodes as well as their relevant decisions of the decision tree. Each node of the tree is written in a separate line. Below is an example of a node:

*1395 0.91798 SumBQ> 329 VarPos> 118,520 AveBQ> 16.4222 AveMQ> 47.1515 \.*

*Depth> 87.9315 AveAS> 68.8356 Ratio> 0.818494 DeltaL>− 0.0100506 \.*

*AveAS> 93.4468 AveMQ> 69.9913 AveBQ> 23.6107 VarPos> 320,454 \.*

*AveAS> 123.286 AveMQ> 71.1475.*

The first item is the number of candidate variants in this node. The second item is the qual value of this node. And the rest items of this line record the decision process that ends up with this node.

## Conclusions

We proposed a decision-tree-based method Fuwa for fast calling variants. Although decision tree is not a very sophisticated algorithm, Fuwa is expected to achieve good performance with regard to accuracy, recall and speed simultaneously. The results of applying Fuwa to a well-studied sample from 1000 Genomes met our expectations on whole-genome sequencing data, whole-exome capture data and low-coverage data. Comparison between high-coverage and low-coverage WGS call sets demonstrates that Fuwa is capable of handling sequencing depth insufficiency, benefiting from the usage of dbSNP and the self-adaption property of machine learning algorithms. Further experiments on 4 samples that have not been used to populate dbSNP added more evidence to Fuwa’s specificity on calling common and rare variants, and the runtime records suggested that Fuwa is not only a fast caller, but also a resource-conserving programme, making Fuwa a competitive choice in processing NGS data that are getting larger every year.

One advantage of machine learning algorithms is that their working parameters do not rely on user settings. Among those popular callers such as SAMtools, there exist many parameters for setting thresholds. Although most parameters have default values that usually work fine, these values are mostly obtained empirically, and when applied to unusual data sets such as low-coverage sequencing data, they are not as useful as they are in common situations. In contrast, Fuwa can automatically learn to adapt to different datasets and keep performing well. We believe that Fuwa is a good choice for significantly improving the throughput of the NGS data analysis pipeline for both high-pass and low-coverage data.

## Abbreviations

AveAS: Average alignment score
AveBQ: Average mapping quality
AveMQ: Average mapping quality
BAM: Binary sequence alignment/map format
BiasStr: Bias of strands
CART: Classification and regression tree
dbSNP: The single nucleotide polymorphism database
EBD: Effective base depth
GATK: Genome analysis toolkit
GIAB: Genome in a bottle
gnomAD: The genome aggregation database
Indel: Insertion and deletion
NGS: Next-generation sequencing
PoorMQR: Poor mapping quality ratio
SAM: Sequence alignment/mapping format
SNP: Single nucleotide polymorphism
SNV: Single nucleotide variant
SumBQ: Sum of base quality
VarPos: Variance of position
VarStr: Variance of strands
VCF: Variant call format
WGS: Whole-genome sequencing
WorMQ: Worst mapping quality
